# SIRT1 activation promotes energy homeostasis and reprograms liver cancer metabolism

**DOI:** 10.1186/s12967-023-04440-9

**Published:** 2023-09-15

**Authors:** Benluvankar Varghese, Ugo Chianese, Lucia Capasso, Veronica Sian, Paola Bontempo, Mariarosaria Conte, Rosaria Benedetti, Lucia Altucci, Vincenzo Carafa, Angela Nebbioso

**Affiliations:** 1 Department of Precision Medicine, University of Campania “Luigi Vanvitelli”, Vico De Crecchio 7, 80138 Naples, Italy; 2 Biogem, Molecular Biology and Genetics Research Institute, Via Camporeale, 83031 Ariano Irpino, Italy; 3 IEOS CNR, Via Sergio Pansini 5, 80131 Naples, Italy

**Keywords:** Liver cancer, SIRT1, SCIC2.1, PGC1α, Energy metabolism, Stress response

## Abstract

**Background:**

Cancer cells are characterized by uncontrolled cell proliferation and impaired bioenergetics. Sirtuins are a family of highly conserved enzymes that play a fundamental role in energy metabolism regulation. SIRT1, in particular, drives many physiological stress responses and metabolic pathways following nutrient deprivation. We previously showed that SIRT1 activation using SCIC2.1 was able to attenuate genotoxic response and senescence. Here, we report that in hepatocellular carcinoma (HCC) cells under glucose-deprived conditions, SCIC2.1 treatment induced overexpression of SIRT1, SIRT3, and SIRT6, modulating metabolic response.

**Methods:**

Flow cytometry was used to analyze the cell cycle. The MTT assay and xCELLigence system were used to measure cell viability and proliferation. In vitro enzymatic assays were carried out as directed by the manufacturer, and the absorbance was measured with an automated Infinite M1000 reader. Western blotting and immunoprecipitation were used to evaluate the expression of various proteins described in this study. The relative expression of genes was studied using real-time PCR. We employed a Seahorse XF24 Analyzer to determine the metabolic state of the cells. Oil Red O staining was used to measure lipid accumulation.

**Results:**

SCIC2.1 significantly promoted mitochondrial biogenesis via the AMPK-p53-PGC1α pathway and enhanced mitochondrial ATP production under glucose deprivation. SIRT1 inhibition by Ex-527 further supported our hypothesis that metabolic effects are dependent on SIRT1 activation. Interestingly, SCIC2.1 reprogrammed glucose metabolism and fatty acid oxidation for bioenergetic circuits by repressing de novo lipogenesis. In addition, SCIC2.1-mediated SIRT1 activation strongly modulated antioxidant response through SIRT3 activation, and p53-dependent stress response via indirect recruitment of SIRT6.

**Conclusion:**

Our results show that SCIC2.1 is able to promote energy homeostasis, attenuating metabolic stress under glucose deprivation via activation of SIRT1. These findings shed light on the metabolic action of SIRT1 in the pathogenesis of HCC and may help determine future therapies for this and, possibly, other metabolic diseases.

**Supplementary Information:**

The online version contains supplementary material available at 10.1186/s12967-023-04440-9.

## Introduction

Healthy cellular function depends on several metabolic activities fueled by nutrient availability, and cancer cells undergo various metabolic changes during tumor progression and metastasis. Metabolic reprogramming is a hallmark of cancer cells and is necessary to meet the energy requirements for rapid cell proliferation and metastasis promotion in nutrient-limited microenvironments [1]. Targeting cancer metabolism has therefore become an increasingly important part of cancer therapy. Post-translational modifications such as acetylation, methylation, glycosylation, and lactylation control metabolic signaling pathways [2]. Sirtuins are a group of class III NAD^+^-dependent histone deacetylases (HDACs) that play a key role in energy homeostasis by interconnecting metabolic networks. Depending on the metabolic state of the cell, sirtuins modulate gene expression by deacetylating histone and non-histone proteins [3]. SIRT1 is a well-studied member of the sirtuin family and is known as a stress-responsive protein deacetylase [4]. Numerous studies have described the potential role of SIRT1 functions in DNA repair, oxidative stress response, metabolism, circadian control, and mitochondrial biogenesis [5–7]. Today, SIRT1 is targeted in many age-related human pathologies, such as cardiovascular disease, diabetes and other metabolic syndromes, chronic inflammation, neurodegenerative disorders, and cancer [8]. SIRT1 has a dual role in tumorigenesis, acting as both tumor suppressor and oncogene depending on the cancer type and stage [8]. SIRT1 expression is drastically reduced in various cancers, including hepatic carcinoma, and its activation inhibits cancer growth [9]. Several studies also report that SIRT1 activation in cancer models results in clinical benefits [10, 11]. In gastric cancer, for example, where low expression of SIRT1 correlates with poor prognosis, SIRT1 activation was able to reverse chemoresistance and cancer stemness via the AMPK/FOXO3a pathway, indicating the potential use of SIRT1 activators as a novel therapeutic strategy in this cancer type [12]. Contrarily, in squamous cell carcinomas, upregulation of SIRT1 is associated with a poor prognosis, as high levels of SIRT1 effectively deacetylate and inactivate p53 [8].

However, the specific function of hepatic SIRT1 in metabolism and cancer is still not fully understood. Over the years, a large body of accumulating evidence suggests that SIRT1 is a key player in both controlling energy status and attenuating metabolic stress [13, 14]. It is well documented that SIRT1 metabolically rewires energy circuits during caloric restriction or fasting [15, 16]. Recently, hepatic SIRT1 has emerged as a critical regulator in liver metabolism and energy homeostasis by altering glycolysis and mitochondrial oxidative phosphorylation (OXPHOS) [16, 17]. Specifically, under glucose-deprived conditions in cancer cells, peroxisome proliferator-activated receptor gamma coactivator 1 alpha (PGC1α) promotes oxidative metabolism to maintain energy homeostasis [18, 19]. SIRT1 is reported to deacetylate PGC1α and promote mitochondrial biogenesis [19, 20]. A recent study also showed that SIRT1 activation using ginsenoside Rc improves energy metabolism via deacetylation of PGC1α and mitochondrial biosynthesis in cardiomyocytes and neurons under physiological and ischemia/reperfusion-injured conditions reducing mitochondrial damage and apoptosis [20, 21]. We previously described the beneficial effects of two SIRT1 activators, SCIC2 and SCIC2.1, in attenuating doxorubicin (DOXO)-induced genotoxic stress and cellular senescence [21]. Given the higher efficacy of SCIC2.1 compared to SCIC2, we recently further investigated its activity in hepatic energy metabolism programs.

Here, we show that hepatic SIRT1 reprograms energy homeostasis under glucose-deprived conditions. Specifically, we found that under glucose deficiency SCIC2.1-mediated SIRT1 activation promotes glucose metabolism, increasing both PGC1α deacetylation and, thus, mitochondrial function and production of adenosine triphosphate (ATP). Surprisingly, SCIC2.1 selectively mediated glucose metabolic adaptations by reducing de novo lipogenesis. Conversely, inhibition of hepatic SIRT1 expression by Ex-527 reversed the effects of SIRT1 activation by SCIC2.1. Our results indicate that hepatic SIRT1 activation under glucose-deprived conditions promotes metabolic reprogramming, thus overcoming nutrient availability.

## Materials and methods

### Cell lines and culture conditions

Human hepatocellular carcinoma (HCC; HepG2), human cardiomyocyte (AC16) and human keratinocyte (HaCaT) cells were purchased from ATCC. Cells were cultured in Dulbecco’s Modified Eagle’s Medium (DMEM; Euroclone) containing 10% fetal bovine serum (FBS; Sigma-Aldrich), 2 mM L-Glutamine (Euroclone), antibiotics (100 U/mL penicillin, 100 mg/mL streptomycin, and 250 mg/mL amphotericin-B; all Euroclone) and maintained with 5% CO_2_ at 37 °C. Cells were grown in serum-free DMEM to induce serum deprivation and were then glucose deprived using low-glucose DMEM containing 5 mM glucose.

### Drugs

SCIC2 and SCIC2.1 were purchased from Enamine and used in all experiments at a final concentration of 50 μM and 25 μM, respectively. DOXO (Pfizer) was used at a final concentration of 0.5 μM. SRT1720 and Ex-527 (Sigma-Aldrich) were used at 5 μM. Dimethyl sulfoxide (DMSO; Sigma-Aldrich) was used to dissolve the drugs.

### Cell viability

A standard MTT assay was performed to determine cell viability, as previously described [22].

### FACS analysis

Cells were collected after pharmacological treatment. After washing with phosphate-buffered saline (PBS) solution (1X PBS, pH 7.4, Microgem), cells were incubated with cycle buffer (1X PBS, 10% NP-40, 10% sodium citrate and 2 mg/mL propidium iodide) for 15 min at room temperature. The cells were then mixed with PI buffer (1X PBS and 2 mg/mL propidium iodide), and the percentage of cell death was determined. Experiments were performed in triplicate and the percentage of the cell population in each cell cycle stage and the proportion of cell death were calculated using a BD FACS Celesta Flow Cytometer (BD Biosciences), as previously described [21].

### Real-time cell proliferation assay

The effect of SCIC2.1 on HepG2 cell was investigated using xCELLigence RTCA system (Roche) as a simple, yet quantitative, method to monitor changes in cell proliferation. This technology measures the flow of electrons between gold microelectrodes in cell plate. The number of adhering cells alters the electron transport and consequently, the impedance. Impedance is than expressed as an arbitrary unit parameter, “Cell Index” (CI) that is related to cellular density and is therefore, an indirect way to evaluate cell confluence. SCIC2.1 was used at 10 μM and 25 μM, and DMSO as a control, and dynamic CI values were monitored at 30-min intervals for 70 h. Calculated CI values were plotted on a line graph. Each experimental condition was tested in triplicate. The reported values correspond to the mean values corrected for the standard deviation.

### Cellular thermal shift assay

Cellular thermal shift assay (CETSA) was carried out as previously described [21]. HepG2 cells were treated with SCIC2.1 at a final concentration of 25 μM, and DMSO was used as a control. After the indicated treatment, cells were trypsinized and washed twice with PBS. Cell pellets were resuspended in PBS (1.5 mL), divided into aliquots (100 μL), and heated for 3 min in a ThermoMixer (Eppendorf) at increasing temperatures (25 °C, 37 °C, 47 °C, and 57 °C) followed by 3 min cooling at 4 °C. At the end of incubation, total protein was extracted, and Western blot analysis was performed.

### SIRT1, SIRT3, and SIRT6 fluorescence assay

A commercial fluorogenic assay kit for SIRT1, SIRT3, and SIRT6 (BPS Bioscience) was used to study in vitro enzymatic activity. A fluorescence assay was performed in 96-well plates (low-binding NUNC black microtiter plate). The final reaction volume (50 μL), composed of 150 mM NAD^+^, 1 mg/mL bovine serum albumin, HDAC assay buffer, 100 μM SIRT1 substrate, 25 ng/μL of the enzyme, and 5 μL of the test sample (SIRT activators/inhibitors) was incubated for 30 min at 37 °C. To stop the reaction, 50 μL of developer assay buffer comprising 70% PBS, 30% ethanol, 10 mM DTT, and 10 mM orthophthalaldehyde (Acros Organics) was added and the mixture was incubated at room temperature. Fluorescence intensities were measured at 350 nm and 460 nm excitation and emission wavelengths, respectively, using an automated Infinite M1000 reader (Tecan). A similar assay was performed for SIRT3 and SIRT6 using different substrates and assay conditions according to the manufacturer’s instructions (BPS Bioscience).

### Counter-screening for nicotinamidase

The tested compounds were assessed in parallel with the presence of a nicotinamidase (NMase) enzyme to potentiate the primary assay, as previously reported [21]. Counterscreening was carried out in a low-binding NUNC black 96-well plate with the same positive and negative reaction controls used in the fluorescence assays for SIRT1, SIRT3, and SIRT6. A final volume of 5 μL of each compound was added to a 5 μL reaction buffer that simulated SIRT1 and to the NMase enzyme mix (NMase-purified enzyme, 1 mM intermediate nicotinamide dilution, and appropriate substrate). The developer buffer was added after 40 min of incubation at 37 °C, and the plate was incubated in the dark for another 30 min at 37 °C. An automated Infinite M1000 Tecan reader was then used to detect the fluorescence.

### Total protein extraction and Western blot analysis

Total protein extraction and Western blot analysis were performed as previously described [22]. Briefly, HepG2 and AC16 cells were collected and washed three times with 1X PBS at 1200 rpm for 5 min at 4 °C. Cell pellets were transferred to a lysis reaction buffer containing 50 mmol/L Tris–HCl pH 7.4, 150 mmol/L NaCl, 1% NP40, 10 mmol/L NaF, 1 mmol/L phenylmethylsulfonyl fluoride, and protease inhibitor cocktail (PIC, Roche). The pellets were vortexed three times every 5 min while kept at 4 °C. The samples were then centrifuged at 13,000 rpm for 30 min at 4 °C, and the protein concentration was determined using Bradford assay from Bio-Rad (California, US). 40 μg of the total protein extract was loaded onto 6%, 8%, 10%, and 15% of sodium dodecyl sulfate–polyacrylamide gel electrophoresis (SDS-PAGE) and subsequently transferred to nitrocellulose membranes. Ponceau red (Sigma-Aldrich) was used to verify that the proteins have been transferred correctly to the filter. The membranes were then blocked with 5% milk, and Tris-buffered saline 1 M with 0.1% Tween (TBS-T) was used for membrane washings. The primary antibody was diluted in TBS-T and incubated at 4 °C overnight. Then, the membranes were washed three times with TBS-T every 5 min, followed by incubation with the horseradish peroxidase-conjugated secondary antibodies diluted in 3% milk (for rabbit and mouse at 1:10,000) for 1 h at room temperature. Immunoreactive protein signals were detected using enhanced chemiluminescence (ECL, Bio-Rad). Primary antibodies were: SIRT1 (#8469), Actyl-p53K382 (#2525), FOXO3a (#12,829), UCP2 (#89,326), LDHA (#3582), PUMA (#98,672), p-ACC (ser79, #3661), ACC (#3676), PDH (#2784), GAPDH (#D16H11), and β-Actin (#4967) all purchased from Cell Signaling Technology (Massachusetts, US); p53 (#sc-126) and α-tubulin (#sc-5286) were purchased from Santa Cruz Biotechnology (Dallas, TX, USA); SIRT3 (#ab264041), SIRT6 (#ab88494), Bax (#ab53154), NOX2 (#ab129068), p21 (#ab109520), SOD2 (#ab68155), and anti-Ac-Lys (#ab61257) were purchased from Abcam (Cambridge, UK); phospho-AMPK (Thr183/172, #E-AB21121) was purchased from Elabscience (Texas, US); PGC1α (#PA5-72,948) was purchased from Thermo Fisher Scientific (Massachusetts, US). All antibodies were used following the manufacturer’s instructions. GAPDH, β-Actin and α-tubulin were used as loading controls. Semi-quantitative analysis was performed using ImageJ software (version 1.44), and the relative intensities are reported in figures.

### RNA isolation and real-time PCR

Total cellular RNA was extracted with Trizol from Invitrogen (Massachusetts, US) after the indicated treatment and time points and converted into cDNA using VILO (Invitrogen), as previously described [22]. cDNA was subjected to qRT-PCR using oligonucleotide primers (100 μmol) obtained from BioFab Research (Rome, RM). All the primers used in this study are reported in Additional file 1: Table S1. The negative control in which cDNA was replaced by water was also included. Signals were normalized to the reference gene, β-actin. Data were then reported as the mean of fold change ± standard deviation (SD).

### Protein extraction and co-immunoprecipitation

HepG2 cells were lysed in RIPA Buffer (1% NP-40, 50 mM Tris–HCl pH 8.0, 150 mM NaCl, 10% glycerol, 1 mM etheylenediaminetetraacetic acid and 1X PIC (Roche) for 20 min on a rotator. The total cell extract was obtained by centrifugation at 13,000 rpm or 30 min at 4 °C, and the protein concentration was quantified by Bradford assay (Bio-Rad). For endogenous immunoprecipitation, 1 mg of protein was incubated with 1 μg of antibody (SIRT1, PGC1α, Ac-Lys, and rabbit IgG (Jackson ImmunoResearch, Cambridgeshire, UK), as a negative control, overnight at 4 °C. A/G PLUS agarose beads (#sc-2003, Santa Cruz Biotechnology) were used to immunoprecipitate the immune-antibody complex for 3 h on a rotator at 4 °C. The beads were washed twice with buffer C containing 0.25% NP-40, twice with PBS buffer containing 0.25% NP-40, and finally twice with 1X PBS. For Western blot analysis, 2X Laemmli sample buffer (360 mM Tris–HCl pH 6.8, 12% SDS, 54% glycerol, 0.01% bromophenol blue) was added to the beads, and 6X Laemmli sample buffer was added to the 10% input. The samples were incubated for 5 min at 95 °C and loaded on a polyacrylamide gel.

### Cellular mitochondrial stress and ATP production

To assess the metabolic status of the cells, we used a Seahorse XF24 Analyzer (Agilent Technologies, Santa Clara, CA, USA) with standard 24-well Seahorse microplates, as previously described [23]. To measure the oxygen consumption rate (OCR) and extracellular acidification rate (ECAR), a Mito Stress Test kit (Agilent Technologies, #103,015) was used after treatment of 6 h with 25 μM SCIC2.1. In detail, 2 × 10^4^ cells were seeded into Seahorse microplates 12 h before analysis. After the adhesion of cells, the medium was replaced with 175 μL of non-buffered DMEM comprising 5 mM glucose, 2 mM glutamine, and 1 mM pyruvate. The cells were then treated with SCIC2.1 at 25 μM for 6 h at 37 °C and subsequently incubated in a CO_2_-free incubator to enable the cells to acclimate to the temperature and pH equilibration before being loaded into the XF24 Analyzer for 100 min. Thus, measurements from T = 0 to T = 100 min were recorded after 6 h of treatment with SCIC2.1. The data were analyzed using Wave software (version 2.2.0, Agilent Technologies). The injection procedure was set up as follows: 1st, oligomycin at a final concentration of 1 μM; 2nd, carbonyl cyanide m-chlorophenylhydrazone at a final concentration of 1 μM; 3rd, rotenone and antimycin A at a final concentration of 1 μM and 0.5 μM, respectively. All the experiments were run in triplicates. A student t-test was used to calculate the p-values, statistical significance was considered as p-value < 0.05, and error bars represent SD.

### Quantification of lipids with Oil Red O staining

HepG2 cells were treated with SCIC2.1 and Ex-527 at 25 µM and 5 µM, respectively, at the indicated time points. The cells were then treated with 4% formalin in PBS for 1 h and rinsed with distilled water at room temperature. The cells were fixed for 5 min in 60% isopropyl alcohol, stained for 15 min at room temperature with a solution of 0.3% Oil Red O in 60% isopropyl alcohol, and then washed with distilled water. The lipid content was extracted by isopropanol and incubated for 15 min absorbance at 540 nm was measured with an Infinite M1000 microplate reader (Tecan). Lipid accumulation was visualized, and images were acquired using a BioTek Cytation 5 cell imaging multimode reader (Agilent Technologies).

### Statistical analysis

All the data were analyzed using GraphPad Prism 9.4.0, and statistical significance was assessed by one-way ANOVA followed by Dunnett’s multiple comparisons tests. p < 0.05 was considered statistically significant.

## Results

### SIRT1 activation does not affect cell cycle and rescues cells from genotoxic stress

**Fig. 1:**
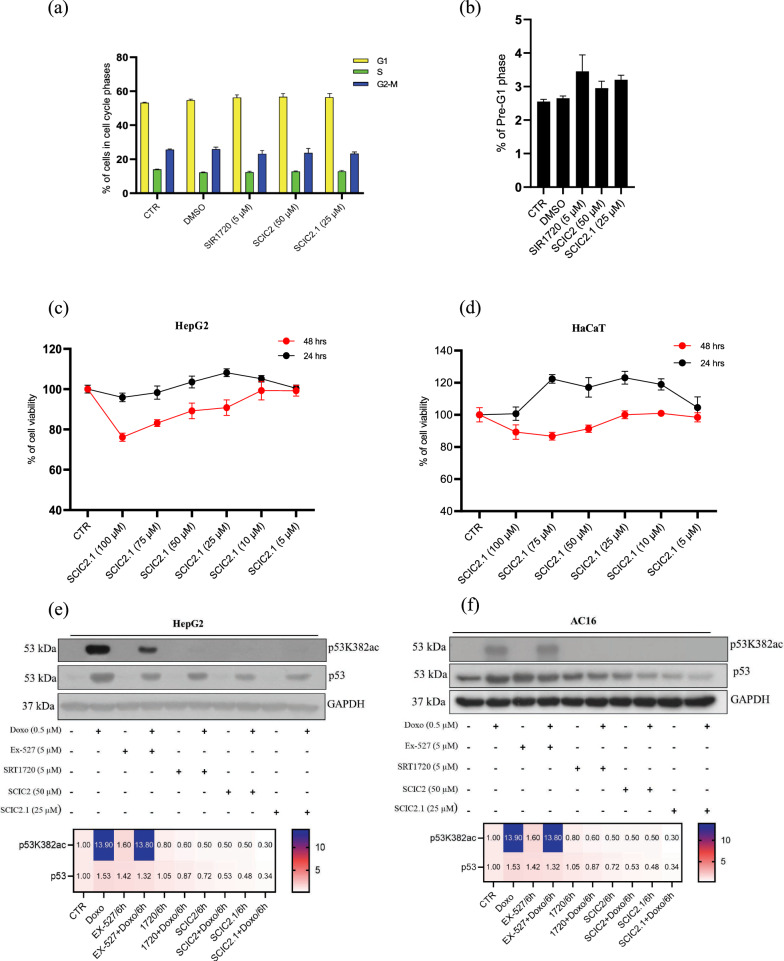
SIRT1 activation does not affect cell cycle progression or cell proliferation and rescues cells from genotoxic stress. **a** Effects of SIRT1 activators on cell cycle progression of HepG2 cells treated with SRT1720 (5 µM), SCIC2 (50 µM), and SCIC2.1 (25 µM) for 24 h. **b** Percentage of cells in pre-G1 phase. **c** and **d** MTT assay on HepG2 and HaCaT cell lines treated with different concentrations of SCIC2.1 for 24 h and 48 h. **e** and **f** Western blot analysis on HepG2 and AC16 cells pretreated with the genotoxic drug DOXO (0.5 µM) for 12 h, and then with Ex-527 (5 µM), SRT1720 (5 µM), and SCIC2 and SCIC2.1 (50 µM and 25 µM, respectively) for 6 h. Heat map showing quantification of p53/p53K382ac bands determined by ImageJ software. Error bars represent the standard deviation (SD) of three biological replicates

We previously showed that the SIRT1 activator SCIC2.1 does not affect cell cycle progression of both tumor and normal cells, and potentiates SIRT1-dependent p53 deacetylation upon DNA damage [21]. Thus, initially to corroborate the biological effects mediated by SCIC2.1 HepG2 cells were untreated or treated for 24 h with three different SIRT1 activators, SCIC2 (50 µM), SCIC2.1 (25 µM), and SRT1720 (5 µM). FACS analysis revealed that none of the three compounds significantly modulated cell cycle progression (Fig. 1a) and cell death, measured as a percentage of cells in preG1 phase (Fig. 1b). We also assessed the cytotoxic effects of SCIC2.1 on HepG2 and HaCaT cells and observed no toxic effect in either cell line (Fig. 1c, d). We further investigated the impact of SCIC2.1 in a real-time cell proliferation assay. The graph in Additional file 1: Fig. S1 shows the CI in HepG2 cells acquired over 70 h. At 25 µM concentration, SCIC2.1 led to rapid cell proliferation. At the lower concentration (10 µM), SCIC2.1 was still able to modulate proliferation, unlike the control. SIRT1 is reported to play a vital role in coordinating and attenuating different cellular stress responses [24, 25]. In mammalian cells, SIRT1 prevents stress-mediated apoptosis via deacetylation of p53 at lysine 382 (p53K382ac) and promotes cellular proliferation [26]. To further explore the role of SCIC2 and SCIC2.1 in modulating SIRT1-mediated functions in stress responses, we analyzed their action on p53 acetylation. Western blot analysis for p53K382ac was performed on HepG2 and AC16 cell lines pretreated with the genotoxic drug Doxorubicin (DOXO) at 0.5 µM for 12 h and then untreated or treated with Ex-257 (5 µM), SRT1720 (5 µM), SCIC2 (50 µM), or SCIC2.1 (25 µM) for 6 h. All three SIRT-activating compounds strongly decreased expression of DOXO-induced p53K382ac, strengthening their ability to potentiate SIRT1-dependent p53 deacetylation, reducing DNA damage effects (Fig. 1e, f). Noteworthy, the well-known SIRT1 inhibitor Ex-527 was obviously able to maintain high expression levels of p53K382ac.

Taken together, these results support the hypothesis that SIRT activation does not affect cell growth and regulates genotoxic stress response.

### SCIC2.1 increases SIRT1 expression upon glucose deprivation and binds to SIRT1

**Fig. 2:**
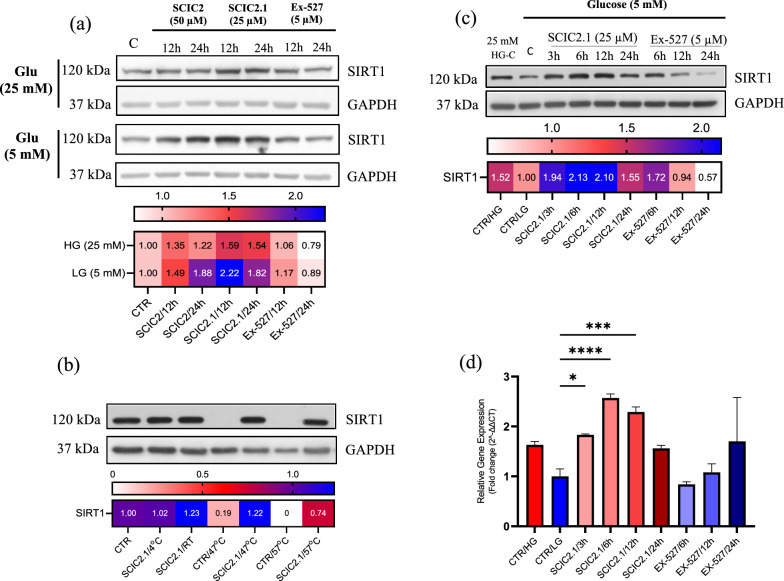
SCIC2.1 increases SIRT1 expression upon glucose deprivation and binds to SIRT1. **a** Western blot analysis of SIRT1 expression in HepG2 cells treated with SCIC2 (50 µM), SCIC2.1 (25 µM), and Ex-527 (5 µM) for 12 h and 24 h under different concentrations of glucose. Heat map showing quantification of SIRT1 bands determined by ImageJ software. **b** CETSA assay performed on HepG2 cells treated with SCIC2.1 at 25 µM and the western blot analyses showing SIRT1 protein expression. Heat map showing quantification of SIRT1 bands determined by ImageJ software. **c** SIRT1 protein expression on HepG2 cells treated with SCIC2.1 (25 µM) and Ex-527 (5 µM) at the indicated time points under low-glucose deprivation. Heat map showing band quantification of SIRT1 protein expression determined by ImageJ software. **d** SIRT1 mRNA fold expression in HepG2 cells after treatment with SCIC2.1 and Ex-527 for the indicated times. Error bars represent the SD of three biological replicates. Statistical significance was calculated using the student *t*-test or one-way ANOVA using GraphPad Prism 9.4.0, and statistical significance is expressed as *p-value < 0.05, ***pvalue < 0.001, ****p-value < 0.0001 vs control

SIRT1-mediated stress response is frequently associated with starving/fasting conditions. During fasting or prolonged glucose deprivation, hepatocytes play a key role in energy metabolism [27], and SIRT1 is involved in regulating hepatic metabolism [28]. We performed a preliminary screening to determine whether SCIC2 and SCIC2.1 enhance SIRT1 expression under conditions of glucose deprivation. We used growth media containing two different glucose concentrations (25 mM and 5 mM) to culture HepG2 cells. As expected, under low-glucose conditions, SIRT1 expression was increased by SCIC2.1 and decreased by Ex-527 (Fig. 2a). These data strongly pointed to SCIC2.1 as a promising candidate for use in our further experiments. We also performed an in vitro enzymatic assay to confirm SIRT1 activation by SCIC2.1. Elevated enzymatic activity was found with SCIC2.1 compared to SCIC2 (158% vs 134%), as in our previous results [21] (Additional file 1: Fig. S2a). No significant NMase modulation by SCIC2 or SCIC2.1 was observed (Additional file 1: Fig. S2a). To better define SCIC2.1 activity, we performed CETSA assay using HepG2 cells to evaluate the interaction between SCIC2.1 and SIRT1 in a physiological cellular environment. Interestingly, compared to the control (DMSO), SCIC2.1 protected SIRT1 from thermal degradation at the maximum temperature of 57 °C (Fig. 2b). We next wondered whether targeting SIRT1 could also attenuate glucose-mediated stress response in HCC cells. We therefore subjected HepG2 cells to prolonged glucose deprivation and found that 48 h of glucose deprivation was able to trigger metabolic stress (Additional file 1: Fig. S3a) and to increase the preG1 phase of the cell cycle (Additional file 1: Fig. S3a). Comparable results were also obtained when HepG2 cells were starved without FBS (Additional file 1: Fig. S3b). We then evaluated whether SCIC2.1 was able to attenuate this stress response by treating HepG2 cells with SCIC2.1 and Ex-527. Upon prolonged glucose deprivation, SIRT1 protein levels were decreased in the control (5 mM glucose vs 25 mM), and treatment with SCIC2.1 and Ex-527 led to SIRT1 upregulation and downregulation, respectively (Fig. 2c). Furthermore, qRT-PCR analysis showed that SCIC2.1 treatment increased SIRT1 mRNA expression (Fig. 2d), supporting our hypothesis that SIRT1 is crucial to attenuate metabolic stress response. The SCIC2.1-mediated activation strategy could therefore be useful to compensate the SIRT1 downregulation effect induced by low glucose.

### SCIC2.1 significantly increases mitochondrial functions via AMPK-p53-PGC1α axis in glucose-starved conditions

**Fig. 3:**
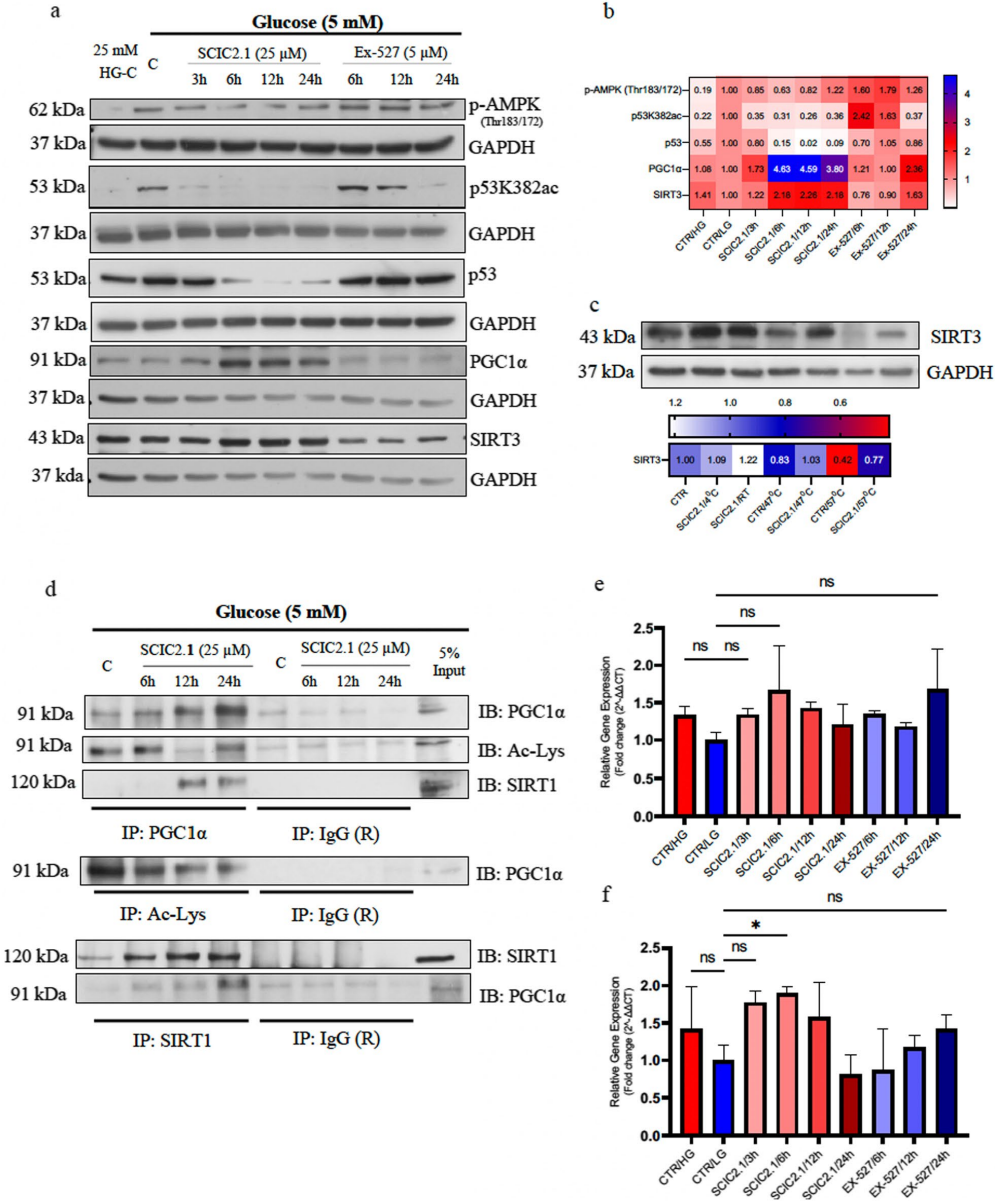
SIRT1 activation by SCIC2.1 significantly increases mitochondrial functions through the AMPK-p53-PGC1α axis upon glucose deprivation. **a** Western blot analysis of pAMPK (Thr183/172), p53K382ac, p53, PGC1α, and SIRT3 in HepG2 cells treated with SCIC2.1 and Ex-527 at the indicated concentrations and time points. Heat map showing band quantification of protein expression. **c** CETSA assay performed on HepG2 cells treated with SCIC2.1 at 25 µM and the western blot analyses showing SIRT3 protein expression. Heat map showing quantification of SIRT1 bands determined by ImageJ software. Heat map showing quantification of SIRT 3 bands determined by ImageJ software. **d** Immunoblots showing the expression of PGC1α, acetyl-lysine and SIRT1 after the treatment with SCIC2.1 (25 µM) at indicated time points. **e** SIRT3 mRNA fold expression and **f** PGC1α mRNA fold expression evaluated after the treatment with SCIC2.1 and Ex-527 at the indicated time points. Error bars represent the SD of three biological replicates Statistical significance was calculated using the student t-test or one-way ANOVA using GraphPad Prism 9.4.0, and statistical significance is expressed as *p-value < 0.05, ^ns^*p*-value > 0.05 vs control

As mentioned above, glucose-mediated starvation upregulates p53 protein levels in an AMPK-dependent manner. AMPK acts as a cellular stress sensor during energy depletion. By Western blot analysis we confirmed that prolonged glucose starvation in HepG2 cells induced AMPK phosphorylation at threonine residues 183/172, and p53 acetylation at lysine 382, compared to cells grown in high-glucose conditions (Fig. 3a). Interestingly, SCIC2.1 was able to strongly decrease the p53K382ac signal, reducing phosphorylation of AMPK by boosting the energy demand (Fig. 3a). These findings suggest that SCIC2.1 exerts an adaptive posttranslational effect in concert with hepatic SIRT1 activation. Several studies report that activation of SIRT1 results in energy homeostasis via mitochondrial biogenesis [29–32]. Since PGC1α is a transcriptional co-activator controlled by SIRT1 and is essential in mitochondrial biogenesis, we investigated its modulation in this experimental setting. Western blot analysis in HepG2 cells maintained in prolonged glucose starvation conditions and treated with SCI2.1 showed upregulation of PGC1α (Fig. 3a) after 6 h of treatment that was maintained until 24 h. In accordance, PGC1α mRNA expression levels were also increased after 6 h-treatment with SCIC2.1 (Fig. 3f). The opposite effects of Ex-527 confirmed the crucial role of SIRT1 activation in mitochondrial biogenesis. Furthermore, there is a strong relationship between the function of SIRT3, another member of the Sirtuin family, and mitochondrial activity. Cellular metabolism is impacted by SIRT3, which is mostly found in the mitochondrial matrix and catalyzes deacetylase reactions on mitochondrial substrates. Aspects of mitochondrial metabolism that SIRT3 specifically controls include the tricarboxylic acid cycle, fatty acid oxidation, OXPHOS, ROS detoxification, mitochondrial dynamics, and the mitochondrial unfolded protein response (UPR). We therefore investigated whether SCIC2.1 was also able to induce expression of SIRT3 in the same experimental setting. Western blot data showed that SCIC2.1 increased SIRT3 protein expression in a time-dependent manner, corroborating the idea that SCIC2.1 acts specifically on mitochondrion-related functions and that it targets an axis in which SIRT1 PGC1α and SIRT3 participate (Fig. 3a). Modulation of mRNA expression also confirmed SCIC2.1-dependent SIRT3 induction (Fig. 3e). In addition, the in vitro enzymatic assay of SIRT3 (Additional file 1: Fig. S2b) provided further evidence that SCIC2.1 is also an activator of SIRT3. To verify the binding action of SCIC2.1 on SIRT3, we also performed CETSA assay using HepG2 cells. Interestingly, compared to the control (DMSO), SCIC2.1 protected SIRT3 from thermal degradation at the highest temperature of 57 °C (Fig. 3c). Together, these results show that SIRT1, SIRT3, and PGC1α contribute to promote mitochondrial biogenesis in response to metabolic stress.

We next sought to better investigate mitochondrial biogenesis via SCIC2.1-mediated deacetylation of PGC1α by immunoprecipitation in HepG2 cells under glucose-starved conditions. To evaluate PGC1α deacetylation, we immunoprecipitated using anti-PGC1α, anti-SIRT1, and anti-Ac-Lys antibodies. Increased expression of SIRT1 by SCIC2.1 in HepG2 cells was associated with upregulation of PGC1α (Fig. 3d). We further examined deacetylation of PGC1α by coimmunoprecipitation with Ac-Lys. In the control cells PGC1α was mainly in acetylated form and the level of the acetylation was decreased by SCIC-2.1 in a time-dependent manner. This finding suggests that SCIC2.1 promotes expression of SIRT1, enhancing its interaction with PGC1α during glucose starvation to promote energy homeostasis. Taken together, these data support the idea that the effect of SCIC2.1 in protecting hepatocytes from metabolic stress caused by glucose starvation depends on SIRT1-PGC1α-mediated mitochondrial function.

### SCIC2.1 promotes ATP production and redox response in glucose-starved conditions

**Fig. 4:**
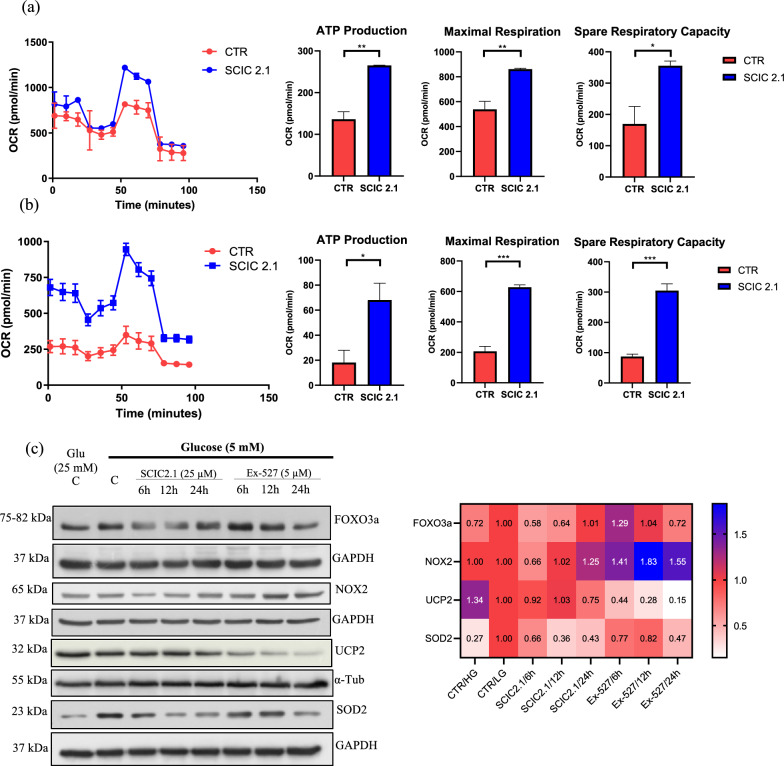
SIRT1 activation by SCIC2.1 promotes ATP production and antioxidant response upon glucose starvation. Mitochondrial respiratory parameters following glucose deprivation and bioenergetic parameters associated with SCIC2.1 treatment, and oxygen consumption rate (OCR) of HepG2 cells: **a** Glucose (25 mM) exposure followed by the treatment with SCIC2.1. **b** Glucose (5 mM) exposure followed by treatment with SCIC2.1. Histograms show the different parameters (ATP Production, Maximal Respiration, Spare Respiratory Capacity). **c** Western blot analysis of FOXO3a, NOX2, UCP2, and SOD2 performed in HepG2 cells treated with SCIC2.1 and Ex-527 at the indicated concentrations and time points. Heat map showing band quantification of protein expression determined by ImageJ software. Error bars represent the SD of three biological replicates. Statistical significance was calculated using the student t-test or one-way ANOVA using GraphPad Prism 9.4.0, and statistical significance is expressed as *p-value < 0.05, **p-value < 0.01, and ***p-value < 0.001 vs control.

SIRT1 is known to orchestrate metabolism and biosynthesis, acting as a key regulator in liver metabolism [17, 30, 33]. Given that SCIC2.1 can promote energy homeostasis via mitochondrial biogenesis, we further explored the effect of SCIC2.1 on ATP production and OCR in HepG2 cells cultured under glucose-starved conditions. Noteworthy, treatment with SCIC2.1 at 25 µM for 6 h promoted ATP production, subsequently inducing maximal mitochondrial respiration capacity in HepG2 cells under low-glucose starvation conditions (Fig. 4a, b; Additional file 1: Fig. S4b). Interestingly, when mitochondrial function was completely inhibited by the electron transport chain inhibitor, spare respiratory capacity, and other experimental parameters were similarly improved. Conversely, lower mitochondrial efficiency was observed in HepG2 cells cultured in high-glucose conditions (Fig. 4a, b; Additional file 1: Fig. S4b). Taken together, these findings suggest that SCIC2.1 promotes ATP production and mitochondrial respiration capacity under metabolic stress. These results clearly show that SIRT1 activation by SCIC2.1 metabolically reprograms the cellular energy steady state under metabolic stress.

In general, cancer cells depend on glucose for survival, whereas glucose deprivation induces the generation of ROS and promotes cell death [34]. SIRT1 and SIRT3 are known to regulate ROS production and promote cell longevity and metabolic adaptations [35, 36]. We therefore investigated the promising strategy of using SCIC2.1 to modulate SIRT1-mediated antioxidant regulation. We found that SIRT1 activation by SCIC2.1 regulates the expression of several antioxidant proteins such as FOXO, NOX2, UCP2, and the ROS-detoxifying enzyme SOD2 [3, 37]. Interestingly, we found that FOXO3a was significantly reduced by SCIC2.1 in a time-dependent manner (Fig. 4c). Furthermore, in HepG2 cells, SOD2 was increased in glucose-starved conditions and, surprisingly, SCIC2.1 treatment decreased its expression. NOX2 was early decreased at 6 h while UCP2 was not altered (Fig. 4c). These results are in line with the observed SCIC2.1-mediated increase in ATP production and oxygen consumption (Fig. 4a, b). The opposite effect of the SIRT1 inhibitor Ex-527 was clearly revealed for FOXO3a and NOX2 (Fig. 4c). Together, these results shed light on the role of SCIC2.1 as a protective agent via its activation of SIRT1 under metabolic stress.

### SCIC2.1 alters metabolic programming and inhibits lipogenesis in glucose-starved conditions

**Fig. 5:**
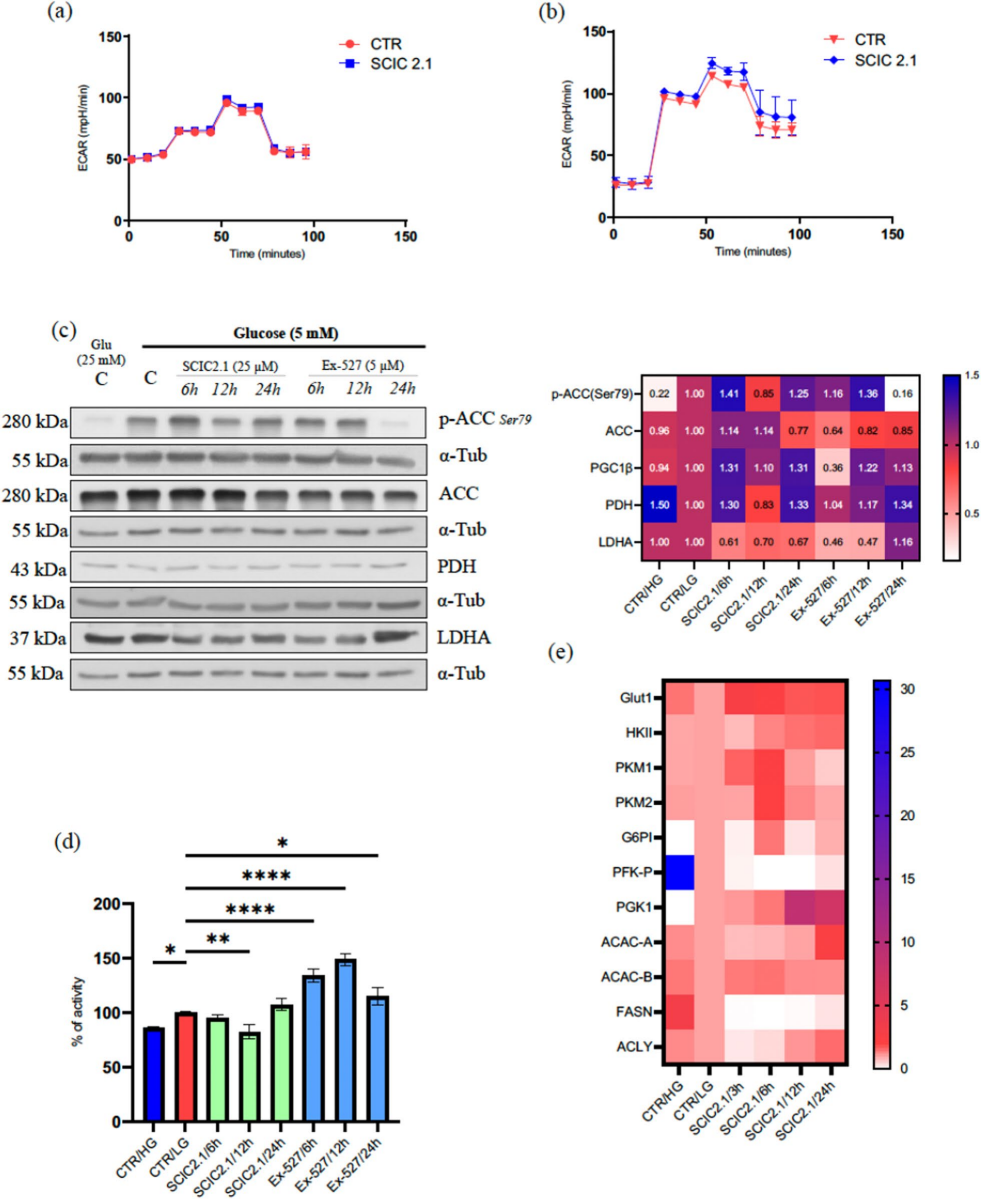
SCIC2.1 alters the metabolic program and inhibits lipogenesis upon glucose starvation. Extracellular acidification Rate (ECAR) of HepG2. **a** Glucose (25 mM) exposure followed by the treatment of SCIC2.1 **b** Glucose (5 mM) exposure followed by the treatment of SCIC2.1. **c** Western blot analyses of SCIC2.1 alters the metabolic program upon glucose starvation: Western blot analyses p-ACC (Ser79), ACC, PDH, and LDHA was performed in HepG2 cells treated with SCIC2.1 and Ex-527 at indicated concentration and time points. Heat map showing the band quantification of protein expression. **d** quantification of Oil Red Oil staining at 540 nm (n = 3) using TECAN in HepG2 cells treated with SCIC2.1 and Ex-527 at indicated time points. **e** Heat map showing different expression levels of genes involved in glycolysis and lipid metabolism in HepG2 cells treated with SCIC2.1 at 25 µM. Statistical significance was calculated using the student *t*-test or one-way ANOVA using GraphPad Prism 9.4.0, and statistical significance is expressed as *p-value < 0.05, **p-value < 0.01, and ****pvalue < 0.0001 vs control. Error bars represent the standard deviation (SD) of three biological replicates

To corroborate our findings, we investigated the role of metabolic targets in reprogramming metabolic adaptations in glucose-starved conditions. We initially assessed the impact of SCIC2.1 on ECAR parameters, including glycolytic activity. SCIC2.1 was moderately able to alter different parameters of ECAR, such as glycolytic capacity and glycolytic reserve, under glucose starvation compared to HepG2 cells cultured in high-glucose conditions (Fig. 5a, b). We then evaluated some of the major metabolic genes involved in glucose and lipid metabolism. As already shown, AMPK phosphorylation is an important activator of lipid metabolism at low energy levels, and SCIC2.1 inhibited its phosphorylation at 12 h of treatment (Fig. 3a). Given that SCIC2.1 can reprogram the metabolic pathway under glucose starvation, we investigated the phosphorylation of acetyl-CoA carboxylase (ACC), a downstream target of AMPK that plays a key role in lipogenesis. SCIC2.1 downregulated phosphorylation of ACC at serine 79 at 12 h, while no alteration in total ACC levels was observed in HepG2 cells (Fig. 5c). At 24 h p-ACC signal was maintained by SCIC2.1 and totally abrogated by Ex-527. Thus, the functional redundancy between SCIC2.1 and energy homeostasis could depend on the SIRT1-PGC1α pathway. To better understand the role of SCIC2.1 in lipid metabolism under conditions of glucose deprivation, we also performed an Oil Red O staining assay to verify lipid accumulation. Oil Red O staining revealed a significant decrease in lipid accumulation after 12 h of treatment with SCIC2.1 in HepG2 cells under glucose starvation (Fig. 5d; Additional file 1: Fig. S5). In contrast, Ex-527 markedly increased lipid accumulation (Fig. 5d; Additional file 1: Fig. S5). These results suggest that during glucose deprivation SCIC2.1-mediated SIRT1 activation leads to the use of lipids as an alternative energy source to drive oxidative metabolism.

We also evaluated the action of SCIC2.1 on glycolytic energetics by Western blot experiments. The two major end products of glycolysis, pyruvate dehydrogenase (PDH) and lactate dehydrogenase A (LDHA), play an important role in metabolic alterations. During glucose deprivation, we found that SCIC2.1 promoted the expression of PDH, leading to the conversion of ACC (Fig. 5c). Surprisingly, a concomitant time-dependent decrease in LDHA was observed in HepG2 cells treated with SCIC2.1 (Fig. 5c). Regardless, these results are compatible with our findings from bioenergetic flux analysis by Seahorse. We also investigated the differential expression of key enzymes involved in glycolysis and lipid pathways to obtain more robust evidence of metabolic alterations. SCIC2.1 treatment at the indicated time points uniquely altered glycolytic genes in response to ATP production. The downregulated expression of lipidic genes such as ACLY and FASN was detected and corroborated the downregulation of phosphorylated ACC and the results of Oil Red O staining (Fig. 5e). Furthermore, Ex-527 abrogated the effects of SCIC2.1, suggesting that SIRT1 inhibition leads to increased lipid and glucose accumulation in HCC cells. Overall, the action of SCIC2.1 stimulates hepatic energy expenditure by reprogramming glucose and lipid metabolism to fuel oxidative energy demands.

### SCIC2.1 indirectly activates SIRT6 and modulates metabolic stress response

**Fig. 6:**
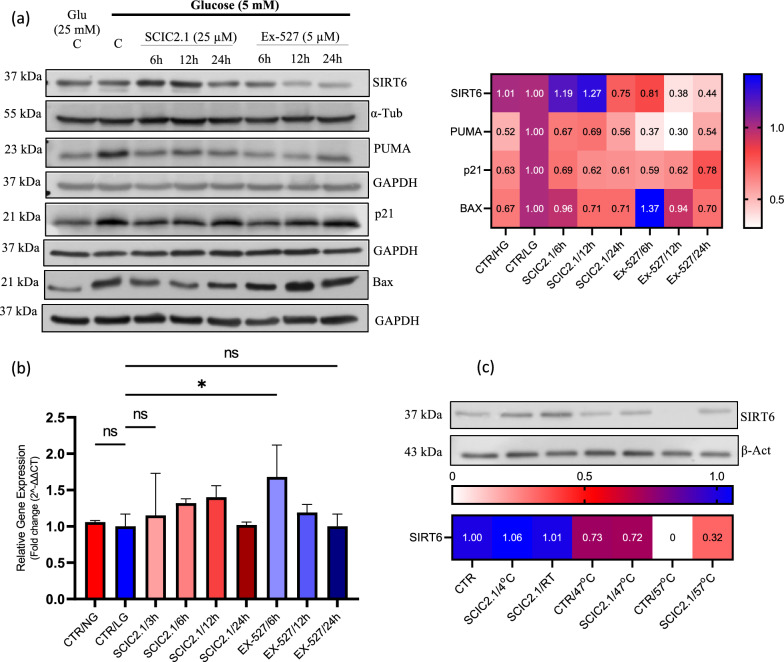
SCIC2.1 indirectly activates SIRT6 and rescues cells from metabolic stress. **a** Western blot analysis of SIRT6, PUMA, p21, and Bax performed in HepG2 cells treated with SCIC2.1 and Ex-527 at the indicated concentrations and time points. Heat map showing band quantification of protein expression. **b** SIRT6 mRNA fold expression evaluated after treatment with SCIC2.1 and Ex-527 at the indicated time points. **c** CETSA assay performed on HepG2 cells treated with SCIC2.1 (25 µM) and SIRT6 protein expression was analyzed. Heat map shows quantification of SIRT6 bands determined by ImageJ software. Statistical significance was calculated using the student *t*-test or one-way ANOVA using GraphPad Prism 9.4.0, and statistical significance is expressed as *p-value < 0.05, ^ns^*p*-value > 0.05 vs control. Error bars represent the standard deviation (SD) of three biological replicates

Since SCIC2.1 modulates hepatic metabolism via the SIRT1-p53-PGC1α axis, we also examined the role of SCIC2.1 in p53 downstream targets. p53 is reported to directly activate SIRT6 expression and to repress gluconeogenesis by inhibiting PGC1α [38, 39]. We assessed whether SCIC2.1 requires SIRT6 to modulate SIRT1- and p53-mediated cellular proliferation under glucose starvation. SCIC2.1 was able to upregulate SIRT6 at 6 h and 12 h, whereas a downregulation was observed in cells treated with Ex-527 (Fig. 6a). To better investigate SIRT6 modulation, we performed an in vitro enzymatic assay. The high activity of the NMase enzyme indicated that there were no SCIC2.1induced changes in SIRT6 enzymatic activity (Additional file 1: Fig. S2c), even if CETSA showed that SCIC2.1 was able to bind SIRT6 (Fig. 6c). Subsequent qRT-PCR analysis also revealed moderate expression of SIRT6 upon treatment with SCIC2.1 under glucose starvation in HepG2 cells (Fig. 6b). These findings could indicate that SCIC2.1 does not activate SIRT6 expression directly but through interaction with p53, which in turn leads to elevated cell proliferation.

Lastly, we explored the ability of SCIC2.1 to attenuate metabolic stress by modulating p21 in a p53-dependent manner. Prolonged glucose starvation induced an increase in p21 in control cells (Fig. 6a). Interestingly, SCIC2.1 reduced the expression of p21 and promoted cell proliferation (Fig. 6a). We also analyzed the expression of p53 pro-apoptotic target genes such as PUMA and Bax. In HepG2 control cells cultured in 5 mM glucose, the expression of PUMA and Bax increased, whereas SCIC2.1 drastically reduced the expression of these targets, leading to increased cell proliferation (Fig. 6a). Together, our findings show that SCIC2.1 can rescue cells from metabolic stress and promote their optimal functioning through SIRT1 gain of expression.

## Discussion

Epigenetic alterations play a pivotal role in energy metabolism, and histone acetylation/deacetylation regulates metabolic intermediates in response to energy deficit. SIRT1 is known to act as a metabolic regulator in response to cellular energy needs. Increasing evidence supports the hypothesis that SIRT1-mediated metabolic adaptations have a beneficial role in glucose homeostasis, mitochondrial biogenesis, inflammation, and apoptosis [40]. We previously reported the promising activity of a novel SIRT1 activator, SCIC2.1, that activates SIRT1 attenuating genotoxic stress by deacetylating p53 at lysine 382 and reducing senescence induction by decreasing senescence-associated beta-galactosidase activity [21]. Starting from this evidence, we here explored SCIC2.1 role in energy metabolism in HCC. The liver is a central metabolic organ that regulates metabolic homeostasis [41]. Liver cancer progression is frequently associated with dysregulation of metabolism. SIRT1 is significantly expressed in HCC cell lines and tumor tissues [42] and facilitates metastasis [43], while pharmacological SIRT1 activation was found to limit cancer growth in mouse models [44]. Using SIRT1 activators such as SRT1720, SRT1460, and SRT3025 was reported to effectively inhibit tumor growth in a xenograft model and to promote the survival of pancreatic cancer cells [45]. However, the effects of SIRT1 as a cancer promoter or a suppressor are still poorly understood.

Our study supports SCIC2.1 as a promising candidate for effectively maintaining the metabolic plasticity of cells. Under conditions of prolonged glucose deprivation, SIRT1 activation by SCIC2.1 leads to metabolic reprogramming in HCC cells. SCIC2.1 induces the expression of SIRT1, thus attenuating metabolic stress by regulating the SIRT1-mediated AMPK-p53-PGC1α pathway. Our FACS analysis revealed that prolonged glucose deprivation induces an increase in the pre-G1 phase of the cell cycle (Additional file 1: Fig. S3a) and a concomitant upregulation of p53 expression (Fig. 3a). SIRT1 is known to deacetylate p53 under cellular stress conditions [46]. Interestingly, we found that upon extended glucose deprivation SIRT1 expression was upregulated by SCIC2.1 (Fig. 2a) and deacetylated p53 at lysine 382 (Fig. 3a), attenuating metabolic stress. Glucose deprivation in hepatocytes is reported to activate AMPK, modulating the expression of PGC1α similarly to SIRT1 [47]. A previous study showed that pharmacological activation of SIRT1 improved glucose metabolism in an AMPK-dependent posttranslational manner [48]. Under glucose deprivation SCIC2.1 reduces phosphorylation of AMPK at Thr183/172 (Fig. 3a) increasing energy demand, and, by Warburg effect, changes glucose metabolism, leading to rapid proliferation (Additional file 1: Fig. S1) [49, 50]. For instance, under nutrient deprivation, cancer cells switch from glycolytic metabolism to OXPHOS in order to meet energy demands [51]. Aerobic respiration strongly depends on mitochondrial function to produce ATP and, intriguingly, SIRT1 deacetylates PGC1α, promoting mitochondrial biogenesis (Fig. 3). Indeed, we found that SCIC2.1 promoted mitochondrial biogenesis by deacetylating PGC1α in a SIRT1-dependent manner (Fig. 3d). SCIC2.1 treatment increases ATP production under glucose deprivation. In contrast, we observed no significant alterations in ATP production and spare respiratory capacity when the cells were cultured in high-glucose conditions (Fig. 4a, b). Our findings, therefore, indicate that the metabolic effect of the SIRT1 activation-mediated pathway plays a crucial role in cellular energy expenditure, supporting published data [20, 52]. Interestingly, under glucose deprivation SCIC2.1 was able to modulate the expression of SIRT3 (Fig. 3a) and other several genes (Fig. 4c) involved in the ROS production [53]. In particular, via SIRT3 activation SCIC2.1 was able to reduce the high expression of SOD2 in cells grown in glucose deprivation conditions, controlling mitochondrial function in a time-dependent manner. These results showed the role of SCIC2.1 on the antioxidant mechanism via the possible activation of SIRT1-SIRT3 functions.

Metabolic reprogramming is a feature of tumor growth, and SIRT1 regulates several genes involved in glucose and lipid metabolism [54]. Evidence in the literature also supports the role of SIRT1 mediated metabolic adaptations in combating metabolic stress [54, 55]. The ECAR response to SCIC2.1 treatment consistently increased glycolytic capacity through the availability of extracellular glucose at an early stage, with a concomitant increase in levels of lactate. At the same time, we observed no significant alterations in HepG2 cells cultured in 25 mM glucose (Fig. 5a, b). Given the potential deacetylation of PGC1α by SCIC2.1 in response to glucose deprivation, we investigated alterations in glucose and lipid metabolism. We observed that SCIC2.1 promotes glycolysis, enhancing ATP production by increasing the expression of PDH, a gatekeeper of glucose oxidation in the early stage. Subsequently, reduced expression of PDH was detected at the late response of SCIC2.1 and prevented the pyruvate metabolite from oxidizing. A clinical study reported that high level expression of LDH in tumors stimulates anaerobic metabolism via increasing glycolysis. SCIC2.1 downregulates the expression of LDHA and reduces lactate production, suggesting its promising anti-cancer activity [56]. Concerning lipid metabolism, SCIC2.1 reduced lipid storage and promoted fatty acid oxidation to regulate energy balance upon glucose depletion. Notably, the reduction in p-ACC at the later response of SCIC2.1 was significantly attenuated with a decrease in phosphorylation of AMPK (Fig. 3a, 5a). Our Oil Red Oil assay and qRT-PCR data of key enzymes of glycolytic metabolism further indicate that the SIRT1 gain of function under nutrient deprivation promotes fatty acid oxidation by inhibiting lipogenesis. It is feasible that a SIRT1-mediated increase in ATP production underlies the induction of metabolic reprogramming under glucose deprivation.

Metabolic stress increases p53 transcriptional activity, modulating stress conditions [57] and transcription of metabolic genes [58]. Our data show that after glucose deprivation, p53 acetylation was reduced by SIRT1 activation (Figs. 2, 3) Some studies also describe the action of p53-mediated functions on SIRT6 activation in the regulation of cell proliferation [31]. Our results indicate that SCIC2.1 promotes SIRT6 protein expression, consistent with increased stability of SIRT1 and p53. These observations support our hypothesis that SCIC2.1 promotes SIRT6 expression via p53-mediated transcriptional activity. Further investigations are required to better understand this mechanism. Lastly, the expression levels of p53 downstream targets such as PUMA, p21, and Bax were reduced upon SCIC2.1 treatment, controlling cell growth and proliferation. These findings provide a crucial insight into the role of the SIRT1-p53-SIRT6 pathway as a critical switch in determining cell fate decisions upon metabolic stress.

## Conclusion

Our study shows that SCIC2.1 can protect HCC cells through activation of SIRT1, enhancing energy homeostasis. Overall, the results demonstrate that SIRT1-SIRT3 interactions are required to promote mitochondrial functions by activating the AMPK-p53-PGC1α pathway under glucose deprivation in HCC cells. Surprisingly, upon prolonged glucose deprivation, SIRT1 activation by SCIC2.1 rescues the cells from metabolic stress via indirect activation of SIRT6-p53 mediated stress response and boosts cell survival. Together, our findings shed new light on how SIRT1 activation acts as a key switch in deciding cell fate in response to metabolic stress.

### Supplementary Information

**Additional file 1: Figure S1.** Cell proliferation upon treatment with SCIC2.1 at the indicated concentrations and times using xCELLigence. **Figure S2.** (a), In vitro enzymatic assay of SIRT1 performed on different SIRT1 modulators at the indicated concentrations, and counter-screening of NMase activity performed as in the SIRT1 assay. (b), In vitro SIRT3 enzymatic assay and counterscreening performed using different SIRT1 modulators at the indicated concentrations. (c), In vitro SIRT6 enzymatic assay performed using different SIRT1 modulators at the indicated concentrations. Statistical significance was calculated using the student t-test or one-way ANOVA using GraphPad Prism 9.4.0, and statistical significance is expressed as **p-value < 0.01, ****p-value < 0.0001. Error bars represent the standard deviation (SD) of three biological replicates. **Figure S3.** (a), cell cycle regulation of HepG2 cells glucose deprived for 24 h, 36 h, and 48 h, and graph showing cells in pre-G1 phase. (b), cell cycle regulation of HepG2 cells serum starved for 24 h, 3 6 h, and 48 h, and graph showing cells in pre-G1 phase. Statistical significance was calculated using the student t-test or one-way ANOVA using GraphPad Prism 9.4.0, and statistical significance is expressed as *p-value < 0.05, ****p-value < 0.0001. Error bars represent the standard deviation (SD) of three biological replicates. **Figure S4.** Mitochondrial respiratory parameters following glucose deprivation and bioenergetics parameters associated with SCIC2.1 treatment, and oxygen consumption rate (OCR) of HepG2 cells. (a), Glucose (25 mM) exposure followed by treatment with SCIC2.1. (b), Glucose (5 mM) exposure followed by treatment with SCIC2.1. Histograms show the different parameters (Basal Respiration, Proton Leak, Coupling Efficiency and Nonmitochondrial Oxygen Consumption). (c), extracellular acidification rate (ECAR) of HepG2 cells. Glucose (25 mM) exposure followed by treatment with SCIC2.1. (d), Glucose (5 mM) exposure followed by treatment with SCIC2.1. Histograms show the different parameters (Glycolytic Capacity and Glycolytic Reserve). Statistical significance was calculated using the student t-test or one-way ANOVA using GraphPad Prism 9.4.0, and statistical significance is expressed as *p-value < 0.05, **p-value < 0.01, ***P < 0.001, ^ns^*p*-value > 0.05 vs control. Error bars represent the standard deviation (SD) of three biological replicates. **Figure S5.** Oil Red O staining of lipid droplet accumulation in HepG2 cells treated with SCIC2.1 (25 µM) and Ex-527 (5 µM) at the indicated time points. Images were acquired using a BioTeK Cytation 5 reader. **Table S1.** List of primers used in this study.

## Data Availability

The corresponding author can provide all the data sets used in this work upon reasonable request.

## Abbreviations

ACC: Acetyl-CoA carboxylase
CI: Cell index
DMEM: Dulbecco’s Modified Eagle’s Medium
DOXO: Doxorubicin
ATP: Adenosine triphosphate
ECAR: Extracellular acidification rate
FBS: Fetal bovine serum
HCC: Hepatocellular carcinoma
HDAC: Histone deacetylase
LDHA: Lactate dehydrogenase A
NMase: Nicotinamidase
OCR: Oxygen consumption rate
OXPHOS: Oxidative phosphorylation
PDH: Pyruvate dehydrogenase
PGC1α: Peroxisome proliferator-activated receptor gamma coactivator 1 alpha
PGK1: Phosphoglycerate kinase 1
PIC: Protease inhibitor cocktail
ROS: Reactive oxygen species
UPR: Unfolded protein response
