# Supplementary material for: SIRT1 activation promotes energy homeostasis and reprograms liver cancer metabolism
Source: J Transl Med. 2023 Sep 15;21:627. doi: 10.1186/s12967-023-04440-9 (PMC10504761; doi:10.1186/s12967-023-04440-9)
Supplement: Supplementary file 1 — Additional file 1: Figure S1. Cell proliferation upon treatment with SCIC2.1 at the indicated concentrations and times using xCELLigence. Figure S2. (a), In vitro enzymatic assay of SIRT1 performed on different SIRT1 modulators at the indicated concentrations, and counter-screening of NMase activity performed as in the SIRT1 assay. (b), In vitro SIRT3 enzymatic assay and counterscreening performed using different SIRT1 modulators at the indicated concentrations. (c), In vitro SIRT6 enzymatic assay performed using different SIRT1 modulators at the indicated concentrations. Statistical significance was calculated using the student t-test or one-way ANOVA using GraphPad Prism 9.4.0, and statistical significance is expressed as **p-value < 0.01, ****p-value < 0.0001. Error bars represent the standard deviation (SD) of three biological replicates. Figure S3. (a), cell cycle regulation of HepG2 cells glucose deprived for 24 h, 36 h, and 48 h, and graph showing cells in pre-G1 phase. (b), cell cycle regulation of HepG2 cells serum starved for 24 h, 3 6 h, and 48 h, and graph showing cells in pre-G1 phase. Statistical significance was calculated using the student t-test or one-way ANOVA using GraphPad Prism 9.4.0, and statistical significance is expressed as *p-value < 0.05, ****p-value < 0.0001. Error bars represent the standard deviation (SD) of three biological replicates. Figure S4. Mitochondrial respiratory parameters following glucose deprivation and bioenergetics parameters associated with SCIC2.1 treatment, and oxygen consumption rate (OCR) of HepG2 cells. (a), Glucose (25 mM) exposure followed by treatment with SCIC2.1. (b), Glucose (5 mM) exposure followed by treatment with SCIC2.1. Histograms show the different parameters (Basal Respiration, Proton Leak, Coupling Efficiency and Nonmitochondrial Oxygen Consumption). (c), extracellular acidification rate (ECAR) of HepG2 cells. Glucose (25 mM) exposure followed by treatment with SCIC2.1. (d), Glucose (5 mM) exposure [file 12967_2023_4440_MOESM1_ESM.pdf]

Additional file 1: Figures

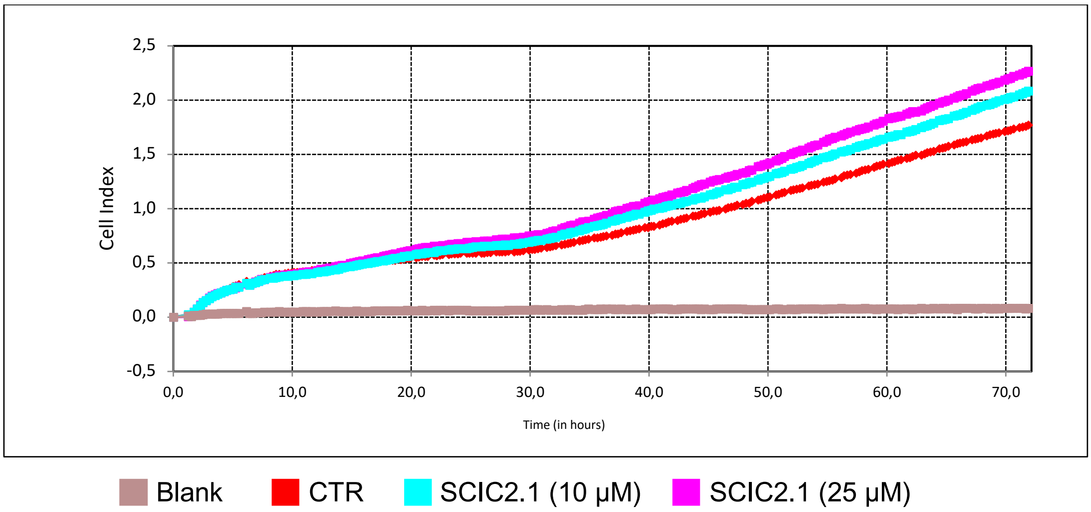

FIGURE S1 Cell proliferation upon treatment with SCIC2.1 at the indicated concentrations and times using xCELLigence.

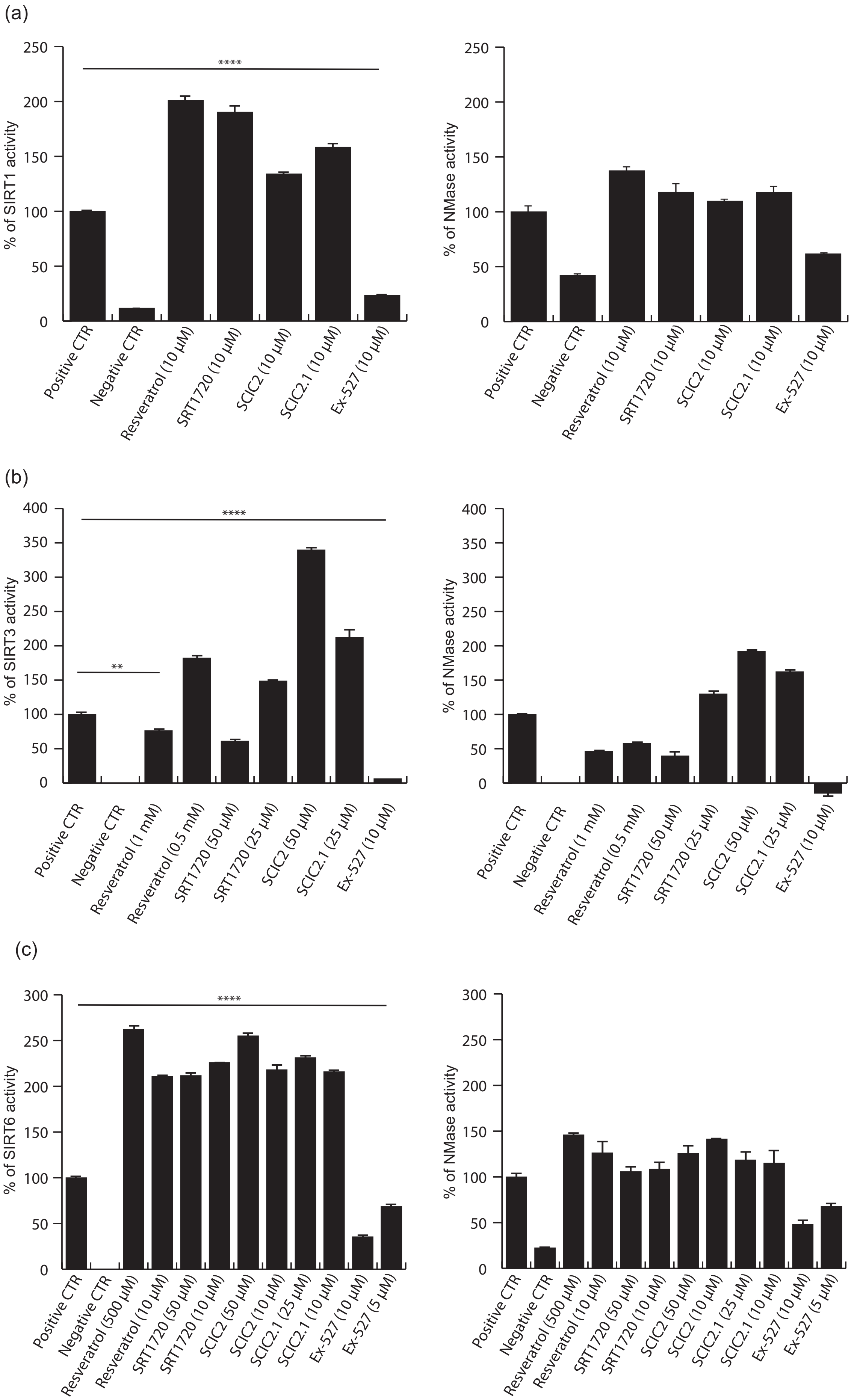

(a)
% of SIRT1 activity
****
0
50
100
150
200
250
Positive CTR
Negative CTR
Resveratrol (10 μM)
SRT1720 (10 μM)
SCIC2 (10 μM)
SCIC2.1 (10 μM)
Ex-527 (10 μM)
% of NMase activity
(b)
% of SIRT3 activity
****
**
300
350
400
Resveratrol (1 mM)
Resveratrol (0.5 mM)
SRT1720 (50 μM)
SRT1720 (25 μM)
SCIC2 (50 μM)
SCIC2.1 (25 μM)
(c)
% of SIRT6 activity
Resveratrol (500 μM)
SCIC2.1 (10 μM)
Ex-527 (5 μM)

FIGURE S2 (a), *In vitro* enzymatic assay of SIRT1 performed on different SIRT1 modulators at the indicated concentrations, and counter-screening of NMase activity performed as in the SIRT1 assay. (b), *In vitro* SIRT3 enzymatic assay and counter-screening performed using different SIRT1 modulators at the indicated concentrations. (c), *In vitro* SIRT6 enzymatic assay performed using different SIRT1 modulators at the indicated concentrations. Statistical significance was calculated using the student t-test or one-way ANOVA using GraphPad Prism 9.4.0, and statistical significance is expressed as **p-value < 0.01, ****p-value < 0.0001. Error bars represent the standard deviation (SD) of three biological replicates.

(a)

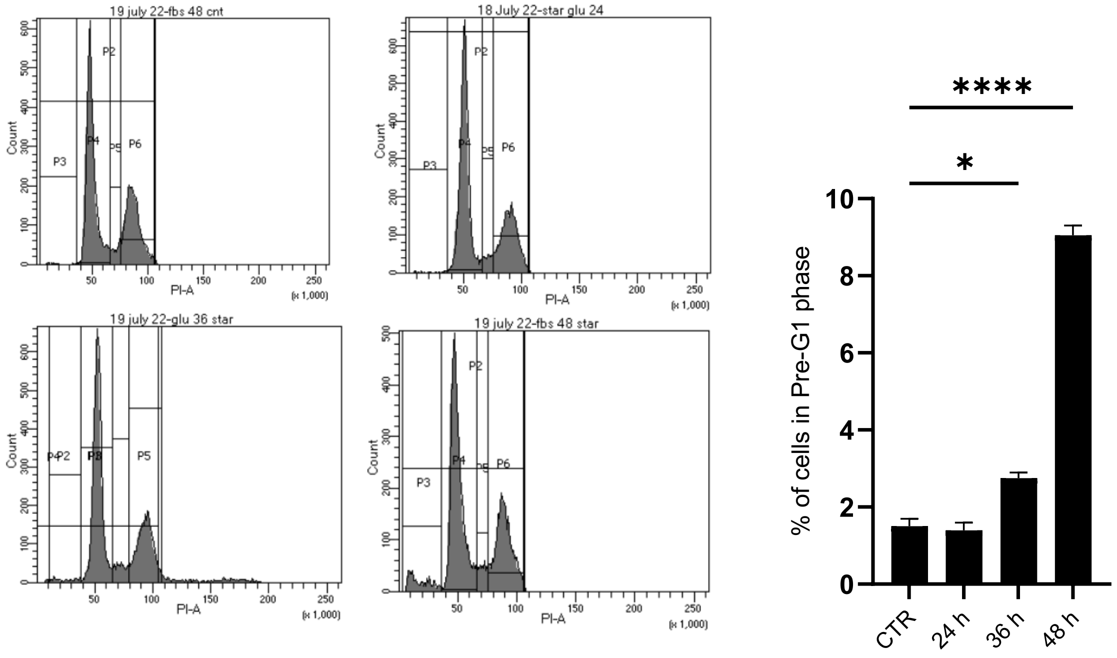

(b)

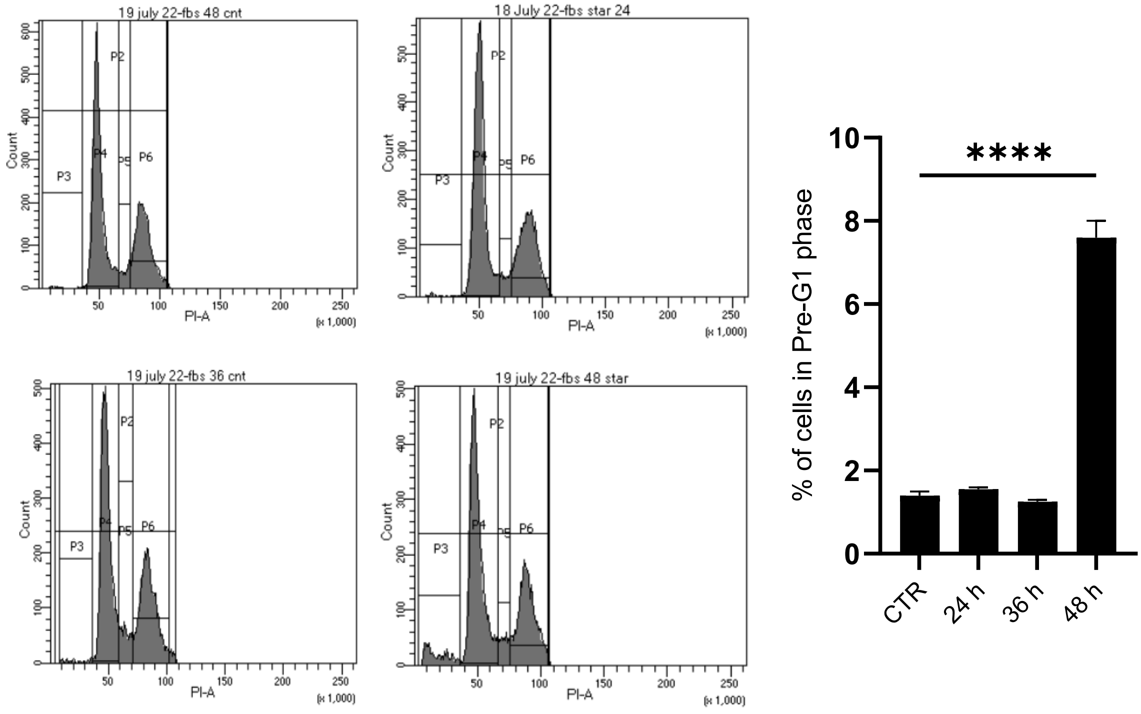

FIGURE S3 (a), cell cycle regulation of HepG2 cells glucose deprived for 24 h, 36 h, and 48 h, and graph showing cells in pre-G1 phase. (b), cell cycle regulation of HepG2 cells serum starved for 24 h, 3 6h, and 48 h, and graph showing cells in pre-G1 phase Statistical significance was calculated using the student t-test or one-way ANOVA using GraphPad Prism 9.4.0, and statistical significance is expressed as *p-value < 0.05, ****p-value < 0.0001. Error bars represent the standard deviation (SD) of three biological replicates.

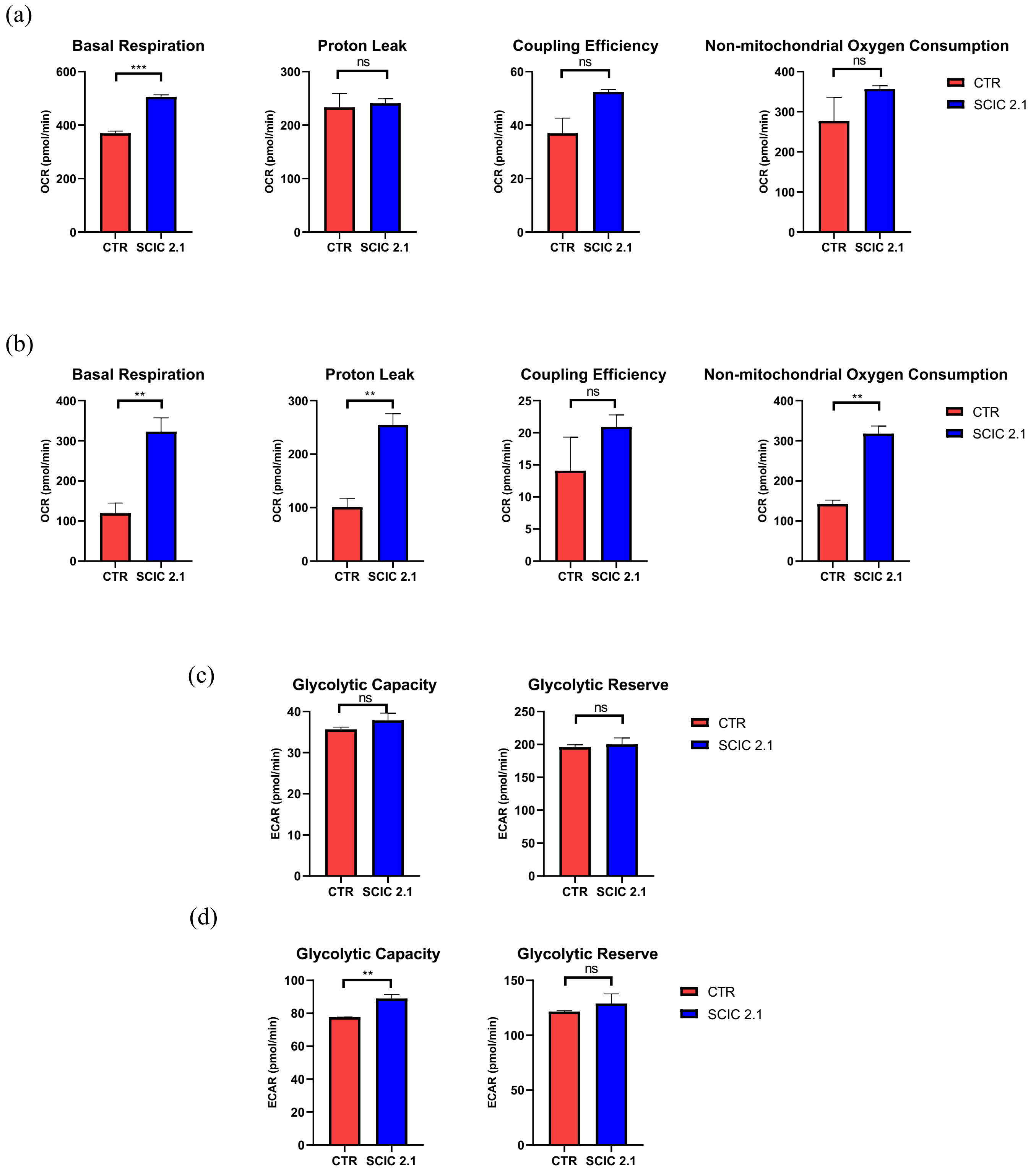

FIGURE S4 Mitochondrial respiratory parameters following glucose deprivation and bioenergetics parameters associated with SCIC2.1 treatment, and oxygen consumption rate (OCR) of HepG2 cells. (a), Glucose (25 mM) exposure followed by treatment with SCIC2.1. (b), Glucose (5 mM) exposure followed by treatment with SCIC2.1. Histograms

show the different parameters (Basal Respiration, Proton Leak, Coupling Efficiency and Non-mitochondrial Oxygen Consumption). (c), extracellular acidification rate (ECAR) of HepG2 cells. Glucose (25 mM) exposure followed by treatment with SCIC2.1. (d), Glucose (5 mM) exposure followed by treatment with SCIC2.1. Histograms show the different parameters (Glycolytic Capacity and Glycolytic Reserve). Statistical significance was calculated using the student t-test or one-way ANOVA using GraphPad Prism 9.4.0, and statistical significance is expressed as $*p$-value < 0.05, $**p$-value < 0.01, $***P < 0.001$, ^{ns}p-value > 0.05 vs control. Error bars represent the standard deviation (SD) of three biological replicates.

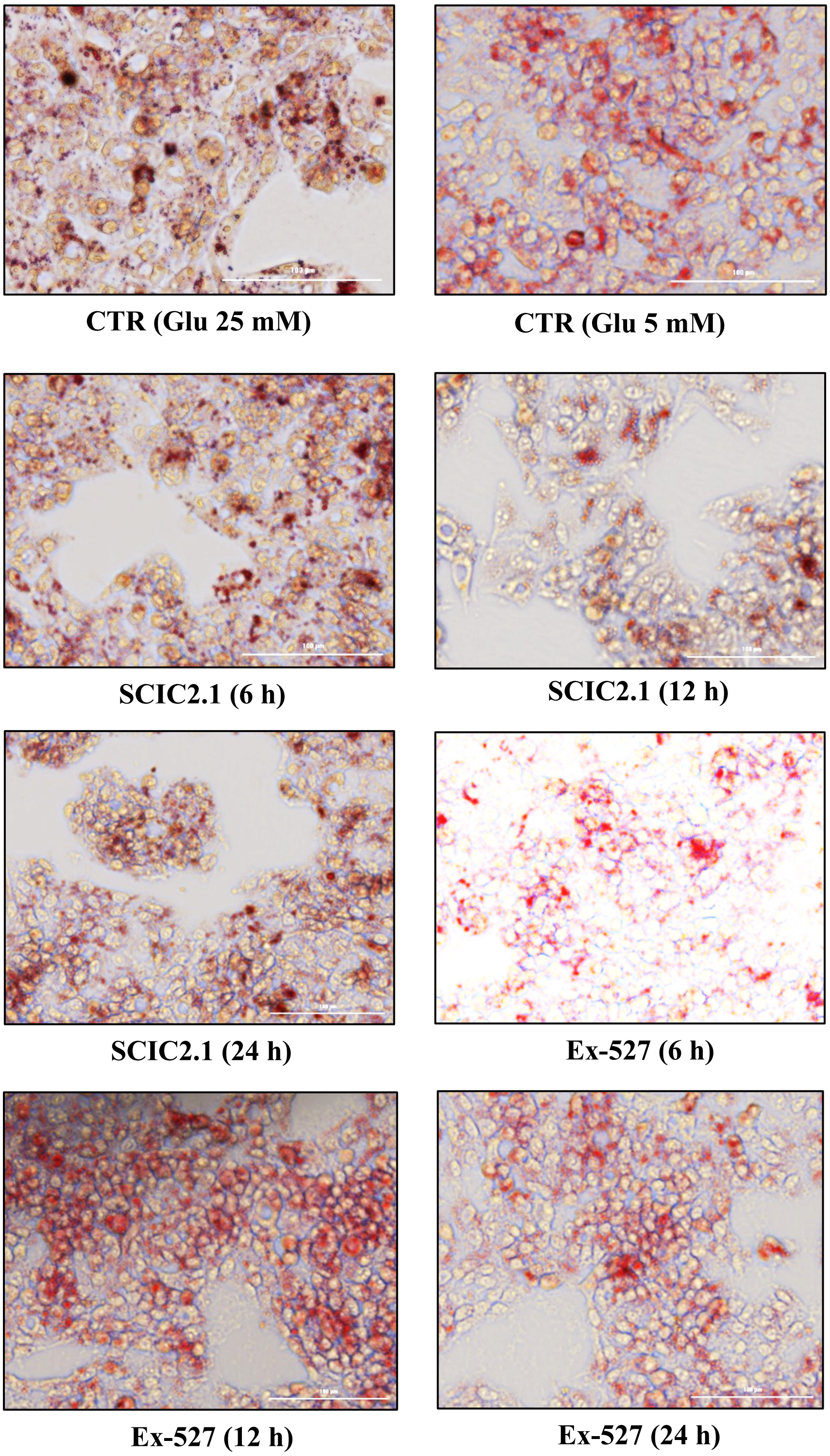

FIGURE S5 Oil Red O staining of lipid droplet accumulation in HepG2 cells treated with SCIC2.1 (25 µM) and Ex-527 (5 µM) at the indicated time points. Images were acquired using a BioTeK Cytation 5 reader.

Gene	Forward (5'–3')	Reverse (5'–3')
SIRT1	GCCTCACATGCA AGCTCTAGTGAC	TTCGAGGATCTGTGCCAATCATAA
SIRT3	TGACTGGTAGGGCTGTGTTT	GAGCTTGCCGTTCAACTAGG
SIRT6	AGGATGTCGGTGAATTACGC	CCAGTTCCCACACCTTCC
PGC1α	AGCGCCGTGTGATTTATGTC	TGCGTCCACAAAAGTACAGC
GLUT1	CTTTGTGGCCTTCTTTGAAGT	CCACACAGTTGCTCCACAT
HK II	GATTTCACCAAGCGTGGACT	CCACACCCACTGTCACTTTG
PKM1	CTATCCTCTGGAGGCTGTGC	CCATGAGGTCTGTGGAGTGA
PKM2	GGGTTCGGAGGTTTGATG	ACGGCGGTGGCTTCTGT
G6PI	AGGCTGCTGCCACATAAGGT	AGCGTCGTGAGAGGTCACTTG
PFKP	CATCGACAATGATTTCTGCGG	CCATCACCTCCAGAACGAAG
PGK1	CGGTAGTCCTTATGAGCC	CATGAAAGCGGAGGTTCT
ACAC-A	GAGGACTGGGAGGATGGG	TTATCCCCAAACCCAGGCA
ACAC-B	GGGCCTATGAGATGTTCCG	GACTCATGAGAGGTCCTGACC
FASN	GGCCTCATAGACCTGCTGAG	TTTGATGCACTGTCCCCTCC
ACLY	GAACTGAGTCAGACACAGTG	CCGAGTAAAGGACCCACAGT
ACTB	GTGGACATCCGCAAAGAC	AAAGGGTGTAACGCAACTA

TABLE S1 List of primers used in this study.
